# *STORM*: A General Model to Determine the Number and Adaptive Changes of Epithelial Stem Cells in Teleost, Murine and Human Intestinal Tracts

**DOI:** 10.1371/journal.pone.0014063

**Published:** 2010-11-19

**Authors:** Zhengyuan Wang, Paul Matsudaira, Zhiyuan Gong

**Affiliations:** 1 Computation and Systems Biology, Singapore-MIT Alliance, Singapore, Singapore; 2 Department of Biological Sciences, National University of Singapore, Singapore, Singapore; 3 Center for BioImaging Sciences, National University of Singapore, Singapore, Singapore; Universität Heidelberg, Germany

## Abstract

Intestinal stem cells play a pivotal role in the epithelial tissue renewal, homeostasis and cancer development. The lack of a general marker for intestinal stem cells across species has hampered analysis of stem cell number in different species and their adaptive changes upon intestinal lesions or during development of cancer. Here a two-dimensional model, named *STORM*, has been developed to address this issue. By optimizing epithelium renewal dynamics, the model examines the epithelial stem cell number by taking experimental input information regarding epithelium proliferation and differentiation. As the results suggest, there are 2.0–4.1 epithelial stem cells on each pocket section of zebrafish intestine, 2.0–4.1 stem cells on each crypt section of murine small intestine and 1.8–3.5 stem cells on each crypt section of human duodenum. The model is able to provide quick results for stem cell number and its adaptive changes, which is not easy to measure through experiments. Its general applicability to different species makes it a valuable tool for analysis of intestinal stem cells under various pathological conditions.

## Introduction

The intestinal epithelium represents the most rapidly renewing tissue in mammals [1]. It has been estimated that billions of cells are exfoliated and replaced in the human intestine on a daily basis [2]. The stem cells play a pivotal role in this process [3] and their deregulation will lead to development of cancer, which is becoming a leading killer in modern society [4], [5]. Analysis of the changes in the intestinal stem cell number upon occurrence of any intestinal lesions would thus serve an important role. Up to date, a general tool is not available for people to analyze the stem cell number and their adaptive changes under different physiological and pathological conditions. This work aims to develop such a tool that facilitates the analysis of stem cells in the intestinal tracts of different species.

Current literature contains multitude of reports studying the epithelium turnover process [6], [7], [8], [9], [10], [11], [12] by using either a grid model [13], lattice-free model [7] or discrete multi-compartmental model [9]. Epithelium migration, cell insertion or apoptosis has been studied in these reports. For example, Gerike *et al* studied dynamics of epithelium proliferation and differentiation, where all columnar cells may become clonogenic stem cells depending on the level of a hypothetical growth factor [6]. Michor *et al* used probablity-based linear models to study the dynamic effects of gene mutations in tumorigenesis [14]. Then d'Onofrio *et al* proposed a non-linear model and suggested that fluctuations in cell death would render the exponential growth of cells irriversible [15]. Johnston *et al* utilized both an age-structured model and a continuous model to study epithelium homeostasis and found that mutations in either death, differentiation or renewal of stem cells or transit amplifying cells will initiate tumorigenesis in the colon [11]. None of the models in current literature, however, was designed to address the number of intestinal stem cells and their adaptive changes.

In this work, a two-dimensional model has been developed to examine the number of intestinal stem cells present in each two-dimensional section of mammalian intestinal crypt, or inter-villus pocket region of teleost intestines, taking input information gained from experimental measurements. This is taking advantage of the important fact that the intestinal epithelium renewal along the crypt-villus axis is essentially a two-dimensional process [16], [17], [18]. It has been our aim to devise a simple and novel model that requires minimal experimental input to directly address the stem cell number. It has been named *STORM* model (STem cell mediated Optimal Renewal of epithelium Model). As an illustration, the model is applied to zebrafish, murine and human intestines, though it may also be applied to other animal models. As the results suggest, the stem cell number is largely conserved across species despite differences among these animal models. In the mean time, the analogy of intestinal epithelium renewal paradigm from zebrafish to mouse and human has rendered zebrafish as an alternative model for study of intestinal stem cells [19], [20], [21], [22].

## Results

### Development of the model

The model was developed based on two assumptions: (1) Epithelial tissue was renewed in a *stem cell – transit amplification – differentiation – apoptosis* paradigm; (2) The epithelial renewal dynamics naturally evolved to have optimal restitutive efficiency.

**Figure 1:**
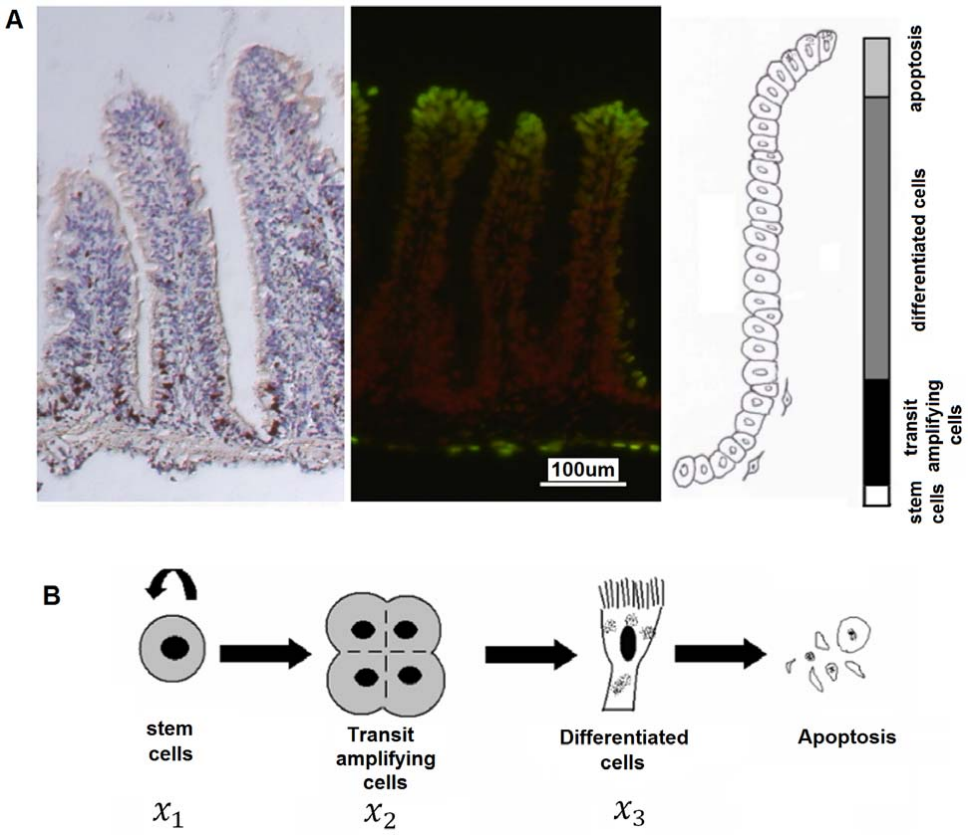
The paradigm of epithelium renewal in the intestine. (A) Cell proliferation and apoptosis in the intestine of zebrafish. Left panel: Cell proliferation assay with proliferating cells stained dark brown. Middle panel: Cell apoptosis assay with apoptotic cells stained green. Right panel: Compartmentalization of epithelium into stem cells, transit amplifying cells, differentiated cells and apoptotic cells. (B) The intestinal epithelium is divided into four components while constructing the model, based on the analogous paradigm of epithelium renewal across teleost, murine and human species. Stem cells maintain their own population through self-renewal, and in the mean time, they produce progenies that will differentiate later on. Transit amplifying cells are directly derived from stem cells and go through rapid expansion. Then they go for cell differentiation and finally apoptosis. Denotation: 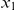
- population of stem cells; 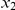
- population of transit amplifying cells; 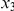
- population of differentiated cells. Note that all populations are normalized against their homeostatic populations in the model.

Take zebrafish as an example. Proliferation assay based on incorporation of bromodeoxyuridine was carried out for zebrafish intestine. Results showed that cell proliferation was restricted in the lower part of villi (Figure 1A, left panel). As the cells migrated upward, they differentiated along either an absorptive or a secretory fate to perform specialized functions. Once they reached the tips of villi, they went through cell apoptosis, as shown by the apoptosis assay (Figure 1A, middle panel), and were then exfoliated. Based on these results, four compartments might be identified along the villus axis, as illustrated in Figure 1B (right panel). In other animals including mouse and human, the intestinal epithelium was organized and renewed in essentially the same manner [23]. Thus, our model was built on the general paradigm of *stem cell – transit amplification – differentiation – apoptosis* for intestinal epithelium, which was applicable to both teleost and mammalian intestinal tracts.

Evidence for natural optimization of epithelial renewal dynamics comes from literature. Mutational analysis of mice heterzygous at the *Dbl-1* locus showed that crypts drift toward monoclonality in the small intestine [17], [24]. Similarly, expression analysis of X-chromosome related gene *G6PD* showed monoclonality of the crypts of large intestine [24]. The mechanism behind these observations was further studied and the concept of neutral competition was clearly proposed recently [25], [26]. For instance, in ref. [26], transgenic mice Lgr5-EGFP-Ires-CreERT2/E-cadherin-mCFP and R26R-Confetti multicolor Cre-reporter were utilized for lineage tracing in the intestine. This novely invented multicolor tracing technique proved that descendants of stem cells constantly went through neutral competition that drived all crypts toward monoclonality in a few months (75% crypts monoclonal in 2 months and 100% in 6 months). Ultimately, descendants of a particular stem cell with the optimal renewal efficiency won out while others disappeared. These results led us to employ an optimization method (to be shown below) to find out the optimal dynamics of crypts as selected by the natural process.

### Workflow of the model

**Figure 2:**
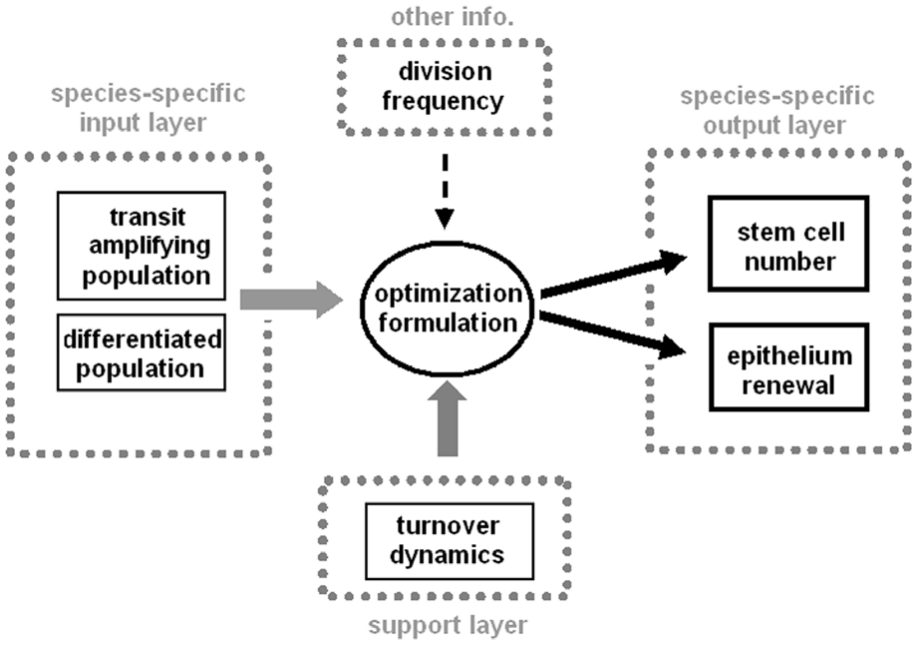
Schematic illustration of the STORM model. The model takes experimental measurement of transit amplifying and differentiated cell populations as input information. By optimizing the turnover dynamics, it yields the number of stem cells required on each section of pocket or crypt of the intestine. It also provides information on epithelium turnover changes, for example, extended turnover cycles due to a reduction in the transit amplifying cells.

The overall workflow of the model is illustrated in Figure 2. Based on the assumptions mentioned earlier and using measured populations of transit amplifying (TA) cells and differentiated cells, the optimization formulation will find out the stem cell number as well as the adaptive changes. Species-dependent outcome of the model would require species-specific input information about the two populations of cells.

### A starting model for epithelium homeostasis

The process of epithelium turnover in the intestine is sketched in Fig. 1B. This model is composed of three components: the stem cells, the transit amplifying cells and the differentiated epithelial cells. The population of stem cells is maintained through self-renewal and production of progenies. The population of transit amplifying cells is maintained through supply from stem cells and expense to cell commitment. The population of differentiated epithelial cells is maintained through supply from transit amplifying progenitors and expense to apoptosis. All the populations are normalized against their homeostatic populations, respectively. Here, the stem cells are defined to be actively involved in TA cell production (instead of remaining quiescent for long periods of time); the TA population is defined to be fast dividing cells that are derived from the stem cells and that are not committed to any lineage yet. Once committed to a particular lineage, either absorptive or secretory, they will be defined as part of the differentiated population.

Based on Fig. 1B, a simple mathematical model can be derived assuming that fluxes of cells move only in a one-way manner. Transit amplifying cells do not reversely dedifferentiate to stem cells (which was suggested a possibility under some special circumstances [23]). Using denotations shown in Fig. 1B, a simple model reads as follows:
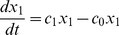
(1)

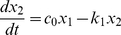
(2)

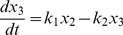
(3)where 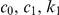 and 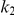 denote the rates of cell flux for the population of stem cells, transit amplifying cells and differentiated cells, respectively. It is worth noting that here we define the transit amplifying cells as fast dividing cells that are derived from the stem cells and they are not committed to any lineage yet. Those lineage-committed cells will become part of the differentiated cells.

A non-trivial steady state may occur only if c_1_ = c_0_. If c_1_>c_0_, the model exhibits exponential growth (unbounded growth of stem cells); whereas if c_1_<c_0_, the model exhibits exponential decay (extinction of stem cells and finally, of everything). Thus the stability of this system depends on whether the relation c_1_ = c_0_ holds and the system is structurally unstable. Biological disturbances may easily lead to unbounded growth of cells. In order for the system to maintain tissue homeostasis in a robust manner, as is observed in the real world, it is necessary to incorporate a feedback mechanism into the model.

### The feedback mechanism in epithelium homeostasis

In view of the tight regulation on stem cells by various signals from both epithelial and mesenchymal cells [27], the marginally stable equation (1) hardly captures the homeostatic feature of the stem cells [28]. Equation (1) may be modified to become structurally stable based on the assumption that stem cell differentiation is related to the second order of stem cell population. Thus equation (1) becomes:
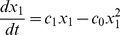
(4)Now the stem cell population may be maintained in a more robust way, but this model still yields limited information about dynamics of the epithelium turnover process. Then a nonlinear term 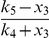 is incorporated into equation (2) and (3), introducing a saturable feedback to stem cell self-renewal and transit amplifying cell division [29], [30], [31], [32], [33], [34], [35], [36], [37], [38].

In the mean time, a factor 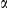, denoting the ratio of transit amplifying population over stem cell population, and a factor 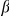, denoting the ratio of differentiated population over transit amplifying population, were incorporated into the model, respectively. To reflect the amplifying nature of the transit population, a factor 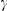 is incorporated. Accordingly, the two modified equations of (2) and (3) now read as follows:
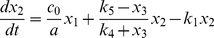
(5)

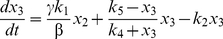
(6)The two nonlinear terms have been introduced with biological support and they signify an important difference between our model and previous models.

For euqation (5), the nonlinear term represents a link between the TA population and the differentiated population. The link has been demonstrated in mice genetically deficient in *Muc2* (C57BL/6J×129/SvOla *Muc2−/−*), a mucin gene expressed only in differentiated cells of the intestine, where impaired cell differentiation via *Muc2* led to spontaneous development of adenomas along the entire gastrointestinal tract [39], [40], a pathology where excessive cells remained proliferative. Similarly, through manipulation of Notch signaling, excessive cell proliferation was observed, accompanied by impaired cell differentiation in the intestine [41]. Conversely, excessive production of differentiated cells was observed, which was accompanied by a reduction in proliferative cells in the intestine, through utilization of Rosa-Notch/Cre+ mice [42]. These examples illustrate the inherent link between populations x_2_ and x_3_ and mathematically, which is modelled by the nonlinear term in equation (5).

For equation (6), the nonlinear term represents a self-fine-tuning mechanism of the differentiated population. Biologically, it has been known that there is certain level of overlap between transit amplifying (fast dividing) cells and lineage committed cells in the intestine. By utilizing the *Math1^beta-gal/beta-gal^* null mice, Yang *et al* showed that some cells kept on dividing even after lineage commitment, producing an overlapped staining by Ki67 and *lacZ* reporter of these cells (representing the differentiation marker *Math1*) [43], illustrating that these cells formed part of the regulatory mechanism responsible for lineage generation process in a self-fine-tuning manner.

The modified model consists of equations (4), (5), (6). As all cell populations are normalized against their homeostatic values, they are to be 1.0 when the system achieves tissue homeostasis. Thus we have:
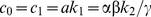
(7)

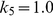
(8)for the homeostatic state. This information will be utilized in the following sections.

### Dynamics of the intestinal epithelium turnover process

The steady state of the system is (1.0, 1.0, 1.0) – normalized against respective cell populations. It represents the homeostatic state of the tissue. Equations (5) and (6) are of special interest as they contain the information on dynamics of epithelium turnover. By setting their gradients to zero, only one non-trivial steady state was found, which is {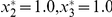}, just as we expected. The Jacobian matrix of for equation (5) and (6) is given as follows:
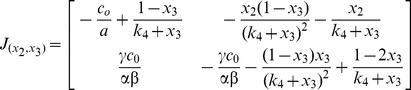
(9)At steady state of {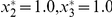}, the Jacobian matrix simplifies as:
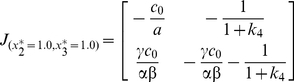
(10)Its eigenvalues are given in two parts. The first part is given by:
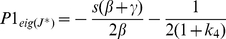
(11)The second part is given by:
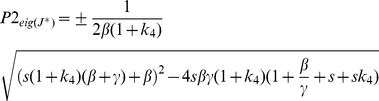
(12)where 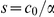. So the two eigenvalues are given by P1+P2. The two eigenvalues have negative real part and the system is locally stable. Upon perturbations, they may re-establish homeostasis with different dynamics, depending on the parametric values (ie. organ-dependent and species-dependent).

### The *STORM* formulation to estimate the epithelial stem cell number

Following our second assumption on optimal restitutive efficiency, the number of intestinal stem cells contained on each section of crypt or inter-villus pocket may be determined by solving the formulation:
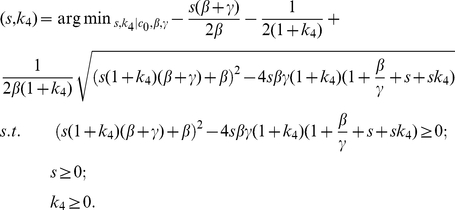
(13)This is a two-dimensional, multi-variate optimization problem with nonlinear objective function and nonlinear constraints. 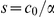 where 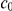 is directly related to the *in vivo* division frequency of the stem cells. 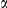 denotes the population ratio of transit amplifying cells over stem cells. 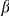 denotes the ratio of differentiated epithelium over transit amplifying progenitors. 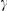 is directly related to the *in vivo* division frequency of the transit amplifying cells. Given the species-specific value of 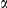, 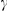 and 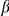, we are able to find out the stem cell number by solving the above formulation.

### General characteristics of the crypt-villus system

**Figure 3:**
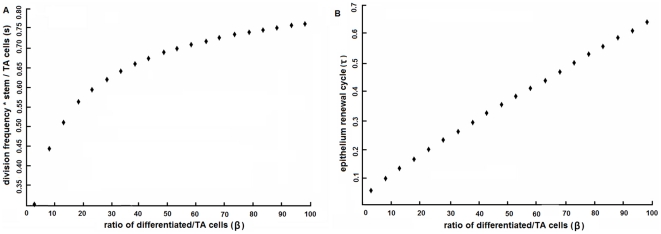
General relationships between 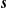, 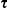 and 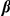. (A) In general, 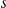 is positively correlated with 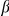. For teleosts where 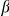 is smaller, 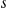 is lower; For humans where 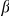 is bigger, 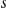 is higher. (B) The epithelium renewal cycle is also correlated to the value of 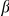. Bigger value of 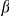 means longer renewal cycle. Cycles are normalized to be dimensionless. 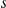: dividing frequency
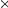
stem population/transit amplifying population; 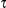: intestinal epithelium renewal cycle; 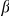: ratio of differentiated epithelium population/transit amplifying population.

There are some general results from the model, which may provide some general knowledge about the crypt-villus system. First, as an adaptive adjustment to the villus size in different species (varying value of 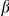), the ratio of stem cell over transit amplifying cell will slightly increase for bigger ratio of 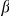 (Figure 3A). This ratio is kept below 0.63 for all 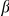 not exceeding 30. For even bigger values of 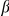, the epithelium renewal process may be excessively slowed down (Figure 3B), rendering a practically non-viable crypt-villus system for the host organism. Second, the renewal cycle of epithelium is correlated to the ratio of differentiated population over transit amplifying population (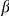). For bigger value of 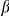, the system needs to support a larger villus size and the epithelium will be renewed at a lower rate. Figure 3B shows the quantitative relationship.

To tailor the model to be species-specific, information about the populations of transit amplifying cells, differentiated cells and *in vivo* dividing frequency of stem cells will be evaluated based on experimental results. The *in vivo* division frequency of intestinal stem cells is not well characterized in the current literature, but it has been speculated to be once or twice every day [44], [45], [46]. For the transit amplifying cells, the amplifying factor 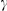 assumes the value of 2.0.

### Determination of the stem cell number in the inter-villus pocket region of zebrafish (*Danio rerio*) intestine

Cell counting over 200 villi in zebrafish based on our own specimens shows the population of proliferating cells (including transit amplifying cells and stem cells) to be 12.5±3.2 cells (mean±std) and the population of differentiated cells with 100±24 cells (mean±std). Representative histological sections are shown in Figure 1A. Based on these data, 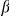 assumes the value of 8.0 for zebrafish.

Formulation (13) may be solved with these parameter values. After obtaining the stem cell number, the population of transit amplifying cells needs to be corrected in order to produce a posteriori-corrected value of 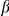. Then the model needs to be solved again. This posteriori-correction process is repeated several times until the solution finally converges and will no longer change. The final solution is as follows:
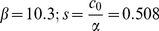
(14)As the population of transit amplifying cells is known from proliferation assays, the number of stem cells may be calculated given the ratio between transit amplifying cells and stem cells. The result is as follows
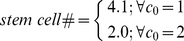
(15)The actual number of stem cells is dependent on their *in vivo* division frequency. If stem cells only divide once per day, there should be 4.1 stem cells present in each inter-villus pocket; if stem cells divide twice per day, there will only be 2.0 stem cells required in each inter-villus pocket. The results are summarized in Table 1.

**Table 1:** Stem cell number in the small intestine of different species as suggested by *STORM* model.

Species	Priori-beta	Posteriori-beta	Stem cell1 division/day	Stem cell2 divisions/day
Zebrafish	8.0	10.3	4.1	2.0
Mice	10.7	16.3	4.1	2.0
Human	23.1	39.0	3.5	1.8

**Figure 4:**
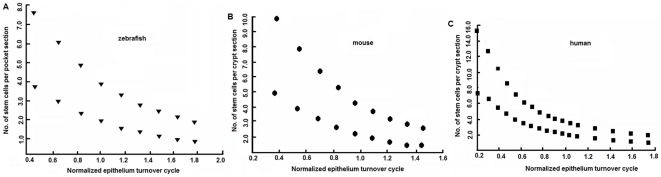
Adaptive changes in the intestinal stem cell number. (A) Intestine of zebrafish. (B) Small intestine of mouse. (C) Duodenum of human. Upper and lower limits of the division frequency of stem cells *in vivo* (once to twice per day) define a range of the number of stem cells required to be present on each section of inter-villi pocket in zebrafish intestine. Reduction in cell proliferation would result in a bigger value of 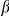 and thus a prolonged epithelium renewal cycle. That would be accompanied by less number of stem cells around. On the other hand, enhanced cell proliferation would result in a smaller value of 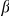 and thus an accelerated epithelium renewal process, accompanied by an increase in stem cell population. That would be the case where hyperplasia or adenoma starts to develop.

**Figure 5:**
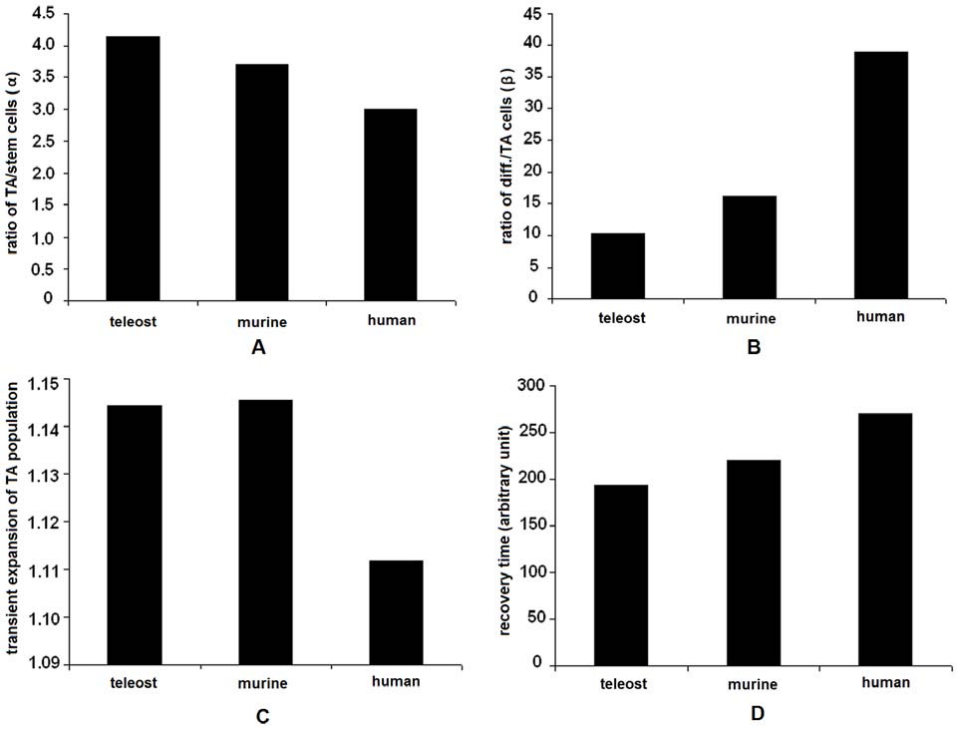
Comparison of epithelium renewal dynamics in different species. (A) The transit amplifying-to-stem cell ratio is the highest in teleost but the lowest in human during normal homeostasis. (B) The differentiated-to-transit amplifying cell ratio is the lowest in teleost but the highest in human during normal homeostasis. (C) As a strategy of efficient tissue restitution, there will be a transient expansion of the transit amplifying population by 10–15% in these species. This value does not vary much as long as the lesion ranges below ∼95% of the epithelium tissue. (D) Recovery time varies in these species. In teleost, epithelium can be restituted in a shorter period of time, but this is achieved by allowing a bigger transient expansion in the transit amplifying population. In human, it takes longer time to complete epithelium restitution, but this is achieved with a tighter mediation over the expansion of the transit amplifying population. These data suggest that these species employ different strategies in maintenance of homeostasis. Compared with intestines of other species, human intestine harbors minimum number of stem cells to support a larger villus size and restitutes epithelium through tightly mediated proliferation to maintain genome integrity and minimize the possibility of carcinogenic transformations.

To examine the adaptive changes in the number of stem cells, the epithelium homeostasis was reduced by 50%, simulating occurrence of intestinal lesions causing damage to the differentiated epithelium. The system responds by initiating tissue restitution process. In the beginning stage, the value of 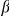 starts at 4.0, the epithelium renewal cycle is 36% faster than the normal cycle and this will trigger an expansion in the stem cell pool and there will be 3.9 to 7.7 stem cells per pocket region (Figure 4A). The expansion of stem cell pool supports a transient expansion of transit amplifying population up to 14.5% (equivalent to one to two cells; Figure 5). As new epithelium are being generated, the ratio of 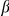 gradually grows back to normal value; The transit amplifying and stem cell population will also return to their respective homeostatic states upon completion of epithelium restitution.

The general correlation between stem cell number and epithelium turnover cycle in zebrafish is shown in Figure 4A.

### Determination of the stem cell number in each crypt of murine small intestine

Proceeding as in the section for zebrafish, we obtained that the population of differentiated epithelial cells is 96±18 in the small intestine of mice; the crypt population is 38±8; the priori-population of proliferating cells (including transit amplifying cells and stem cells) is 11.5±2.5 (the numbers estimated based on references [27], [29], [42], [45], [47], [48], [49]). So 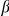 assumes the value of 10.7 for mouse small intestine.

Solve formulation (13) in a priori-posteriori correction manner to have:
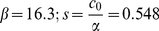
(16)Based on the population of transit amplifying cells, the number of stem cells may be calculated as follows
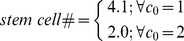
(17)If stem cells only divide once per day, there should be 4.1 stem cells present in each crypt; if stem cells are allowed to divide twice per day, there will only be 2.0 stem cells required in each crypt. Results are summarized in Table 1.

Similar perturbation was conducted to examine the adaptive changes in the number of stem cells in mice. Results of tissue restitution following 50% reduction in differentiated epithelium are shown in Figure 3B, where the epithelium renewed 35% faster than normal and the pool of stem cells expanded from 4.1 to 8.1 per section of crypt (Figure 4B), accompanied by a transient expansion of transit amplifying population up to 14.7% (equivalent to one to two cells; Figure 5C).

### Determination of the stem cell number in each crypt of human duodenum

Proceeding as in the section for zebrafish, we obtained that the population of differentiated epithelial cells in the villus is 120±33; the population of total cells in a crypt is 92±12; the priori-population of proliferating cells (including transit amplifying cells and stem cells) is 8.8±2.1(compiled from refs. [50], [51], [52], [53]). So 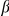 assumes the value of 23.1 for human duodenum.

Solve formulation (13) in a posteriori-correction manner to have:
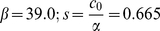
(18)Based on the population of transit amplifying cells, the number of stem cells may be calculated as follows
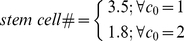
(19)If stem cells only divide once per day, there should be 3.5 stem cells present in each crypt; if stem cells are allowed to divide twice per day, there will only be 1.8 stem cells in each crypt. The results are summarized in Table 1.

Similar perturbation was applied as before. Results are shown in in Figure 3B, where the epithelium renewed 40% faster than normal and the stem cells expanded from 4.3 to 8.6 per section of crypt (Figure 4C), accompanied by a transient expansion of transit amplifying population up to 11% (equivalent to one cell; Figure 5C).

### Comparison of the intestines of different species

To compare the epithelium renewal paradigm among three different species, the ratios between stem cells, transit amplifying cells and differentiated cells are plotted in Figure 5A&B. There is a higher transit amplifying-to-stem cell ratio in teleost. It is the lowest in human accompanied by a higher differentiated-to-transit amplifying cell ratio. This probably reflects two different strategies in the epithelium renewal mechanism: Rapid repair and quick restitution of epithelium take higher priority in the teleost system, whereas relatively slower tissue repair and restitution are allowed in human, with achievement of high fidelity in genomic duplication and reduction in susceptibility of carcinogenic transformations.

**Figure 6:**
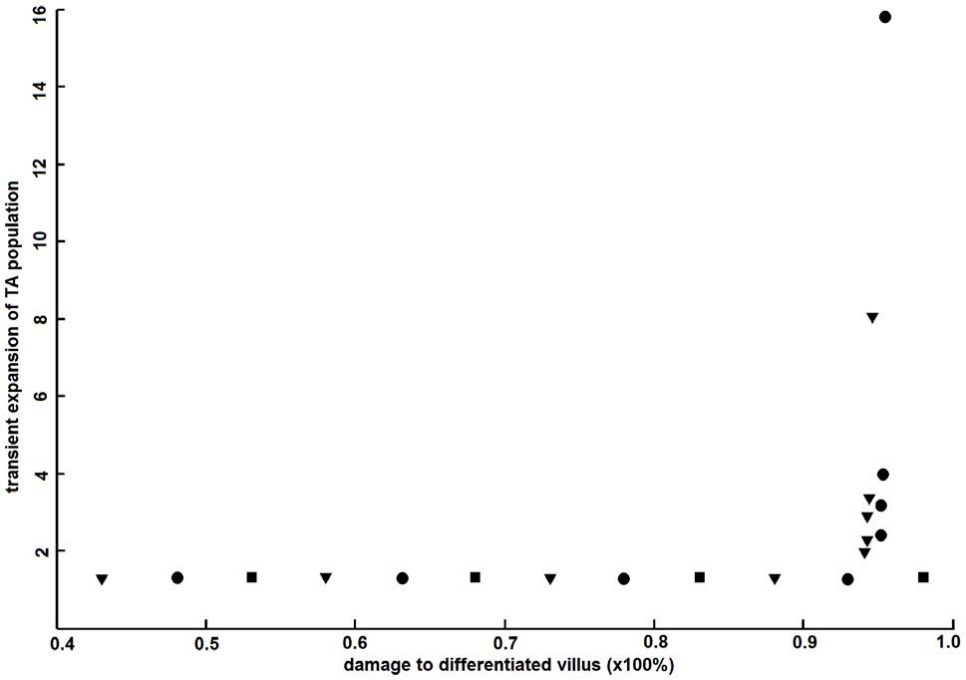
Changes in cell populations during epithelium restitution. The transit amplifying population will transiently expand during epithelium restitution. In the case of extreme tissue lesion where more than 90% tissue is damaged, there will be an overwhelming response of the crypt-villus system and the transit amplifying population will expand in an uncontrolled manner, producing intestinal hyperplasia or adenoma in the teleost and murine intestines, though it seems less likely in human intestine. Denotation: ▾ for zebrafish; • for mouse; ▪ for human.

The process of tissue restitution takes relatively longer time in human, but the transit amplifying population is better restrained from excessive expansion compared with murine and teleost models (Figure 5C&D). This is important as unrestrained expansion of transit amplifying population will lead to development of cancer. As the model reveals, that may happen during epithelium restitution in teleost and murine models, but it is less likely in human intestine (Figure 6).

### Application of the model to help evaluate hyperplasia in human duodenitis and ulcer

Previously, Bransom *et al* reported of mucosal cell proliferation in the duodenum with duodenitis or ulcer in endoscopic biopsies [51]. They intended to find out the presence of epithelium hyperplasia. That may be achieved by quantitative analysis using this model. Based on the histological results, the villi were shortened by 30–50% in duodenal ulcer and duodenitis. Epithelium proliferation, as indicated by the labeling index (the ratio of labeled nuclei to total nuclei in the crypt) is 15.6±1.7 in duodenal ulcer and 17.8±1.5 in duodenitis. Utilizing these data, the model yields that: (1) For duodenal ulcer, 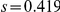, 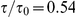, stem cell = 8 on average (In normal human duodenum, the stem cell number is 1.8–2.7, averaged 4.0 as shown earlier). The chi-test for duodenal ulcer shows that it is significantly different from the healthy duodenum (p<0.003). As the output suggests, there is an increase in the stem cell population and an accelerated epithelium renewal rate (about two-fold faster compared with normal rate), implying duodenal hyperplasia. (2) For duodenitis, 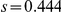, 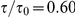, stem cell = 7.5 on average. The chi-test for duodenitis shows that it is significantly different from the healthy duodenum (p<0.02). As the output suggests, there is an increase in the stem cell population and an accelerated epithelium renewal rate (about 1.7-fold faster), implying duodenal hyperplasia. The actual presence of hyperplasia is further evidenced by the histological results of biopsies from the patients, in consistence with analysis result of the current model.

## Discussion

### A novel model for stem cell number in the intestine

In this work, we have devised a novel model that directly addresses stem cell number in the intestine. Utilizing the biological finding of the partial overlap between the transit amplifying population and the differentiated population [1], [43], we introduced nonlinear terms accordingly to model the renewal process in two-dimension. As the intestinal stem cells constantly compete against each other for optimal renewal dynamics [25], [26], the optimization formulation was devised following this philosophy. Solution to the optimal model then allowed us to infer the stem cell number. Design of the model based on the general *stem cells – TA cells – differentiated cells – apoptosis* paradigm has made it possible for the model to be applied to intestines of different species. To our best knowledge, this is the first model of its kind ever reported so far.

### Linear migration of epithelial cells simplifies three-dimensional crypt-villus structures into a two-dimensional model

Though the villi and crypts constitute a three-dimensional inner surface of the intestine, the linear nature of epithelial cell migration [16], [17], [18] nicely simplifies the tissue renewal process into a two-dimensional model. Cell proliferation is restricted near the bottom of crypts (in mammals) or in the inter-villus pocket region (in cryptless zebrafish), whereas apoptosis is restricted at the tips of villi. Epithelium is renewed through cell migration along the villus axis. All cells except the Paneth cells are migrating upward, including columnar cells, goblet cells and enteroendocrine cells in the two-dimensional model.

Differences have been noticed between the two-dimensional systems. In mouse, only a few number of cells are going through apoptosis along each villus (about 7 apoptotic cells over 100 villi [48]). While in contrast, the number of apoptotic cells is notably larger in zebrafish, typically around 15–20 cells per section of villus (Figure 1A). The difference in cell apoptosis agrees with what the model suggests that tissue renewal process goes faster in zebrafish than in mammals (Figure 3B) and in case of tissue recovery, the system recovered more quickly in zebrafish (Figure 5D).

### Achieving optimal epithelium renewal rate is essential to sustainable organ function

The renewal rate of the intestinal epithelium tissue becomes critical in terms of maintenance of tissue integrity, organ function and potential risk of carcinogenic transformation during the life span of the host organism. A high turnover rate would allow quick restitution of the lost tissue due to damage; but on the other hand, high turnover rate would require the presence of more active stem cells around and more frequent cell divisions, increasing the susceptibility to genome duplication-induced mutations and the risk of carcinogenic transformation of the intestinal tissue. These two opposing requirements ultimately lead to optimization of the epithelium turnover rate for a defined organism, allowing maintenance of tissue integrity and organ function with minimal stem cells and cell divisions required. This may be the driving force behind the neutral competition dynamics, and this optimizing procedure persists throughout the adulthood [25], [26]. The optimization model based on this principle has successfully yielded estimates of the stem cell number contained on a section of crypt or inter-villi pocket , and they largely agrees with previous speculations [45], [54].

### STORM model has produced data in general agreement with previous literature

In previous reports, Bjerknes *et al* [16] and Potten [23], [54] estimated that there were 4–6 stem cells in each crypt of mouse intestine (in three dimension). The recent work by Barker *et al* [55], [56], through discovery of stem cell marker Lgr5, showed 6 identifiable stem cells in a section of crypt. Based on their histological results [55], [56], there were approximately 3.5 stem cells per crypt per histological section. Thus in terms of two-dimensional section, our model is able to produce data that generally agree with previous experimental measurements.

As no stem cell marker has been established in zebrafish or human, verification of the model results still awaits future work in this field.

### The number of stem cells appears to be conserved in each pocket/crypt of teleost, murine and human intestines

Despite differences in the intestinal epithelium from teleost to murine, the stem cell number appears conserved within these species. In general, it seems not necessary to maintain a large number of stem cells around from day to day, due to their immortality, sensitivity to DNA damage and carcinogenic potential [57], [58], [59]. In presence of an amplifying mechanism, tissue homeostasis and restitution may be achieved with efficiency by the transit amplifying population without an emergency call on the multipotent stem cells. The human intestine, however, appears to be a more robust system with a more restricted transient expansion in the TA population. This feature may help minimize the potential risk of tumor develpment during the long life-span of humans, compared with teleosts and mice.

### A general model for analysis of stem cell number with equal applicability to teleost, murine and human intestinal tracts

For the first time, a general model is developed to analyze the number of stem cells in the intestinal tracts of teleost, murine and human with minimal requirement of input: mainly information on cell proliferation and differentiation (Figure 2). The fact that the intestinal epithelial cells are essentially renewed in a linear manner [16], [17], [18] has allowed us to develop a two-dimensional model to estiamte the number of stem cells on a section of crypt (or an inter-villi pocket). In absence of a universal stem cell marker for all species, this model provides a useful tool for us to examine the adaptive changes in stem cell number and epithelium renewal dynamics during physiological and pathological states of the organ.

## Methods

The work is approved by Institutional Animal Care and Use Committee (IACUC), National University of Singapore with the approval ID: 070/09.

### Maintenance of zebrafish (*Daino rerio*)

Zebrafish were obtained from local aquarium supply and maintained in a controlled environment according to standard condition with a 14/10 hour light-dark cycle at 28°C [60].

### Histology

Intestines were isolated from euthanized adult zebrafish, washed in ice-cold phosphate-buffered saline (PBS), fixed overnight in a 4% paraformaldehyde solution in PBS at room temperature. Fixed tissue was dehydrated in ethanol with increasing gradients (75%, 90%, 95%, 100% twice), cleared in histoClearII twice and embedded overnight in paraffin that was melted at 58°C. Samples were then sectioned at 7 µM using a Reichert-Jung 2030 machine.

### Immunohistochemistry

25mM Bromodeoxyuridine (Sigma-aldrich, St Louis, United States) was orally administered 50uL per fish 10 minutes before they were euthanized. Immunohistochemistry was performed according to the manufacturer's protocol (cat# 2760, Chemicon International, United States). Briefly, the slides were cleared in histoClear, rehydrated and quenched in 3% hydrogen peroxide, incubated in 0.2% trypsin solution for 10 minutes, denatured for 30 minutes. Slides were subjected to blocking solution for 10 minutes before incubation with detector antibody for 60 minutes at room temperature. Then streptavidin-horse radish peroxidase conjugate was applied for 10 minutes and slides were subjected to a mixture of diaminobenzidine and substrate reaction buffer until color developed. The slides were covered by coverslips and sealed by DePex mounting medium and later, images were taken using a Zeiss Axiovert imaging system.

Immunofluorescent TUNEL assay was carried out according to the manufacturer's protocol (S7111, Chemicon International, United States). Briefly, slides were dewaxed in histoClear, rehydrated and incubated in proteinase K (20 µg/ml) for 15 minutes at room temperature. Equilibration buffer was applied before incubation in terminal deoxyribonucleic transferase enzyme in a humidified chamber at 37°C for 60 minutes. Then stop buffer was applied before slides were incubated in anti-digoxigenin conjugate solution in a humidified chamber for 30 minutes at room temperature in dark. The slides were incubated in 0.5 µg/ml propidium iodide for 10 minutes as a fluorescent counterstaining of nuclei. Finally the slides were covered by coverslips, sealed by DePex mounting medium and images were taken using a Zeiss Axiovert imaging system.
